# Effects of Intranasal and Intravenous Dexmedetomidine on Hemodynamic Responses to Tracheal Intubation and Skull Pin Holder Fixation: A Double-Blinded, Randomized Controlled Trial

**DOI:** 10.7759/cureus.76980

**Published:** 2025-01-05

**Authors:** Garima Singh, Hemlata LNU, Reetu Verma, Aparna Shukla, Premraj Singh, Monica Kohli

**Affiliations:** 1 Anesthesiology, Byramjee Jeejeebhoy Medical College, Ahmedabad, IND; 2 Anesthesia and Critical Care, King George's Medical University, Lucknow, IND

**Keywords:** intranasal dexmedetomidine, intravenous dexmedetomidine, neurosurgical procedures, ramsay sedation scale, skull-pin insertion, α2-adrenoreceptor-agonist

## Abstract

Background

Laryngoscopy and tracheal intubation (L&I) and the fixation of skull-pin head-holders are associated with various sympathetic stimuli leading to hemodynamic changes. These changes may lead to myocardial ischemia, brain edema, an increase in intracranial pressure, or intracranial hemorrhage. Many drugs have been used in different combinations to attenuate the sympathetic responses to L&I and skull-pin insertion. The aim of this study was to compare the efficacy of intranasal (IN) dexmedetomidine with intravenous (IV) dexmedetomidine (IV) in attenuating the hemodynamic responses to L&I and the fixation of skull-pin holders in patients undergoing craniotomy.

Material and methods

This randomized-controlled, double-blind study was conducted on 120 patients with American Society of Anesthesiology (ASA) physical status I and II, aged 18 to 70 years, undergoing elective craniotomy and requiring skull-pin insertion. Patients were randomly divided into two equal groups. Group DIV: IV dexmedetomidine 0.50 µg/kg given over 40 minutes before induction. Group DIN: Undiluted dexmedetomidine 1µg/kg given as IN drops 40 minutes before induction. Heart rate (HR), mean arterial pressure (MAP), systolic (SBP), and diastolic blood pressure (DBP) were noted at baseline and at predetermined intervals after L&I and skull-pin fixation. Sedation scores were assessed preoperatively at baseline and at 10, 20, 30, and 40 minutes after study drug administration. Data were analyzed using IBM SPSS Statistics for Windows, version 25.0.

Results

Both IN and IV dexmedetomidine successfully attenuated the stress responses to L&I and skull-pin fixation without significant hypertension or tachycardia. All hemodynamic parameters (MAP, SBP, DBP, HR) were maintained within normal limits (±20% of baseline) before and during L&I and after skull-pin fixation in both groups.

However, the preoperative Ramsay sedation scale score was significantly higher in the IV than in the IN group at 10, 20, and 30 minutes of drug administration (p<0.05). The incidence of hypoxia and bradycardia was also higher in the IV than in the IN group. Nausea, vomiting, or respiratory depression were not observed in any patient.

Conclusion

Both IN and IV dexmedetomidine are effective in blunting the hemodynamic responses to L&I and skull-pin fixation. However, IN dexmedetomidine is a better alternative to IV dexmedetomidine as it causes less sedation and fewer side effects.

## Introduction

Laryngoscopy and tracheal intubation (L&I) are associated with sympathetic stimulation leading to various hemodynamic changes such as tachycardia and hypertension, and may even lead to myocardial ischemia [1]. The application of a skull-pin holder for stabilizing the head during craniotomy/neurosurgical procedures also produces nociceptive stimulation resulting in abrupt hemodynamic changes under general anesthesia [2]. Many drugs have been used in different combinations to attenuate the sympathetic responses to L&I and skull-pin insertion [3-6]. Other techniques, including oral gabapentin, pin-site infiltration with local anesthetic, and scalp block have also been used to blunt the hemodynamic responses to skull-pin insertion with variable success [7-10].

Dexmedetomidine, a centrally-acting α2-adrenoreceptor agonist, reduces the hemodynamic stress response and plays a role in brain protection [11-13]. However, intravenous (IV) dexmedetomidine produces bradycardia and hypotension, and its sedative effect is more pronounced than its analgesic effect. To avoid these side effects, many alternative routes of dexmedetomidine administration are under trial. Some recent studies have found intranasal (IN) dexmedetomidine to be as effective as IV dexmedetomidine in attenuating hemodynamic responses to L&I [14,15]. However, we have not come across any study comparing the efficacy of IV and IN dexmedetomidine for this purpose in neurosurgical patients or for attenuation of hemodynamic responses to skull-pin fixation. Thus, we designed this study to compare the efficacy of IN dexmedetomidine with IV dexmedetomidine in attenuating the hemodynamic responses to L&I and the fixation of a skull-pin holder in neurosurgical patients undergoing craniotomy. The primary objective of the study was to assess changes in mean arterial pressure (MAP) after L&I and skull-pin fixation. The secondary objectives were to examine changes in heart rate (HR), systolic (SBP), and diastolic blood pressure (DBP), along with sedation scores and other adverse effects.

## Materials and methods

The present randomized controlled study was conducted in the Department of Anesthesiology at King George’s Medical University, Lucknow, after receiving clearance from the Institutional Ethics Committee (118/Ethics/21) and CTRI registration (CTRI/2021/08/035835). A total of 120 cases undergoing elective craniotomy during the study period and meeting the inclusion criteria (patients between 18 and 70 years; American Society of Anesthesiology-Physical Status (ASA Physical Status) I or II; undergoing elective craniotomy requiring skull-pin insertion) and giving informed written consent were recruited for this study. Patients with a history of head injury, previous craniotomy, pregnancy, ischemic heart disease, second- or third-degree heart block, respiratory disease, predicted difficult airway, or known allergy or hypersensitivity to dexmedetomidine were excluded from the study.

Patients were randomly divided into two groups of 60 each using a computer-generated random number table, and the sealed envelope method was used for group allocation: Group DIV (IV dexmedetomidine group) and Group DIN (Intranasal dexmedetomidine group). It was a double-blind study where both the anesthesiologist administering the study drug and the patient were unaware of the group assignment. All study drugs were prepared and coded by a consultant anesthesiologist not involved in patient management or data collection.

A detailed pre-anesthetic check-up was conducted along with appropriate investigations. Patients received premedication with 0.25 mg of tablet Alprazolam the night before surgery and were fasted for 6 hours. On the day of surgery, patients were moved to the preoperative area an hour before surgery. After attaching standard monitors such as pulse oximetry, ECG, and non-invasive blood pressure (NIBP), baseline parameters were recorded, and IV fluids were started. The study drug was administered through intranasal and IV routes at the pre-calculated rate as per group allocation.

Patients in Group DIN received intranasal dexmedetomidine (1 µg/kg) in undiluted form (100 µg/ml) dripped into both nostrils in equal volume using a 1 ml insulin syringe 40 min before induction while in a supine position with head tilted down. Group DIV patients received an equivalent volume of normal saline intranasally. Patients were advised not to sneeze or suck after intranasal administration.

Patients in Group DIV received an IV infusion of dexmedetomidine (0.50 µg/kg) over 40 min from a diluted preparation (4 µg/ml). Group DIN patients received an equivalent volume of normal saline intravenously.

After that, induction of general anesthesia was performed followed by L&I. In the operative room, HR, MAP, SBP, DBP, and oxygen saturation (SpO2) were noted at the following time points: 1) Baseline (DB), 5 mins (D5), 10 mins (D10), 20 mins (D20), 30 mins (D30), and 40 mins (D40) after administering the study drug; 2) Before L&I (LI), at 1 min (I1), 3 mins (I3), and 5 mins (I5) after intubation; and 3) Before pin insertion (PB), at 1 min (P1), 5 mins (P5), 10 mins (P10), 20 mins (P20), 30 mins (P30), and 60 mins (P60) after pin insertion.

Sedation status was assessed using the Ramsay Sedation Scale (RSS) score in both groups at baseline (SB), 10 mins (S10), 20 mins (S20), 30 mins (S30), and 40 mins (S40) after study drug administration [16].

Standard techniques for general anesthesia were used in both groups. In addition to the standard monitors, end-tidal carbon dioxide (EtCO2), invasive blood pressure monitoring, and bispectral index (BIS) were attached in the operative room. Monitoring continued throughout the perioperative period. Patients were preoxygenated with 100% oxygen for 3 min followed by induction with IV propofol (1-2 mg/kg) and fentanyl (2 μg/kg). IV vecuronium bromide (0.1 mg/kg) was administered to facilitate endotracheal intubation. A low-flow anesthesia technique was used (50% O2 - N2O at 1 L/min) along with a propofol infusion and intermittent doses of vecuronium (0.1 mg/kg). Patients were placed on volume-controlled ventilation using a closed circuit. EtCO2 was maintained between 30-40 mmHg. MAP, HR, SBP, and DBP were recorded up to 60 min after study drug administration. Episodes of bradycardia (heart rate <50/min), hypotension (MAP <20% of baseline), and hypoxia (SpO2 <90%) within the study period were noted and treated accordingly. Bradycardia was treated with Inj Atropine 0.6 mg IV. For hypotension, fluid boluses and vasopressors were administered after ruling out and managing factors like excessive depth of anesthesia. Episodes of hypoxia were treated with 100% oxygen.

IV neostigmine (0.05 mg/kg) along with glycopyrrolate (0.01 mg/kg) was administered for reversal of neuromuscular block after surgery completion. After extubation, patients were transferred to the post-operative recovery room and later to the ward. Sedation scores within the study period and other adverse effects were also noted.

Sample size calculation

The sample size was calculated based on the change in MAP after L&I in a previous reference study by Niyogi S et al., using the formula [17]:

 n = 2(Zα+Zβ)2σ2 /(X1-X2)2

Zα = 1.96 at 95% CI

Zβ = 0.841 at 80% power

The sample size was n=60 per group to detect a difference of 10 mmHg in MAP with a 95% confidence interval and 80% power. Extra samples were added to account for drop-outs/exclusions.

Statistical analysis

Data were analyzed using IBM SPSS Statistics for Windows, version 25.0. Comparison of numerical variables between two groups was done by Student’s unpaired t-test for normally distributed data, and by the Mann-Whitney U test for skewed data. Intergroup comparisons were conducted using repeated measures ANOVA followed by Tukey’s test as a post-hoc test if data were normally distributed; for skewed data, Friedman’s ANOVA followed by Dunn’s test was used as a post hoc test. All p-values of less than 0.05 were considered statistically significant.

## Results

**Figure 1:**
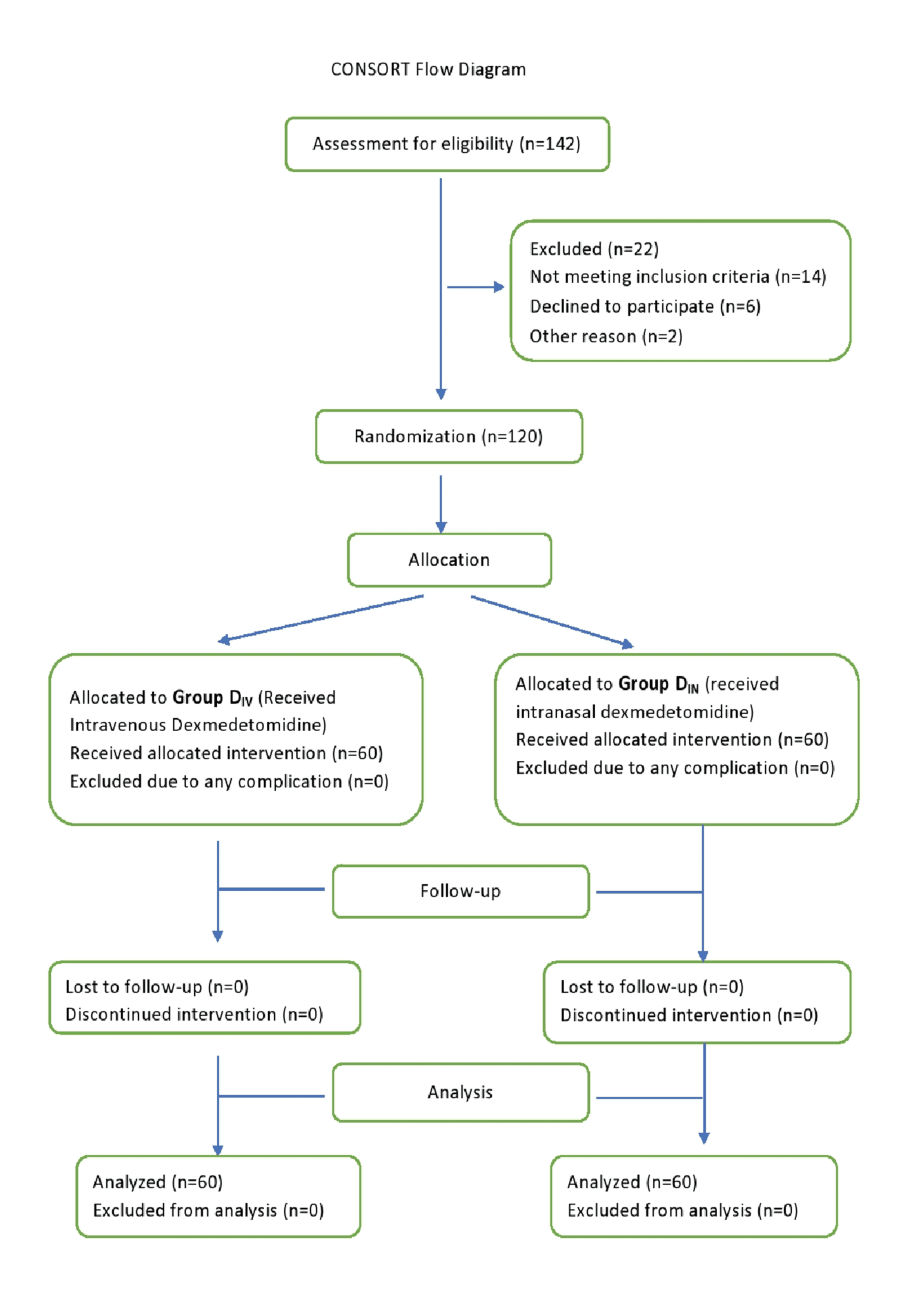
CONSORT flow diagram. CONSORT: Consolidated Standards of Reporting Trials.

Out of the initial 142 patients who were assessed for eligibility, 22 were excluded, and finally, 120 patients participated in this study (Figure 1).

Both groups were comparable in terms of the demographic profile (mean age, gender, and ASA grade) and anthropometric parameters (weight, height, and BMI) of the patients under study (Table 1). Mean MAP, SBP, DBP, HR, and RSS sedation score were comparable at baseline.

**Table 1:** Demographic and anthropometric details of the studied patients. ASA: American Society of Anesthesiologists; M/F: Male/Female. Data expressed as mean ± SD or frequency. n = number of cases; % = percentage of cases. Analyses performed using an independent 't' test and *chi-square test. A p-value < 0.05 was considered significant.

Parameters	Group DIV (n=60)	Group DIN (n=60)	P-value
Age (Years)	43.52 ± 12.27	41.27 ± 12.0	0.312
Gender (M/F)	34/26 (56.7% /43.3%)	36/24 (60.0%/40.0%)	0.712*
ASA (I/II)	37/23 (61.7% /38.3%)	32/28 (53.3%/46.7%)	0.355*
Weight (Kgs)	62.45 ± 5.5	64.23 ± 5.62	0.082
Height (m)	1.59 ± 0.08	1.60 ± 0.07	0.843
BMI (Kg/m^2^)	24.51 ± 2.28	25.2 ± 3.44	0.194

In both groups, there was a decrease in mean MAP, SBP, DBP, and HR from baseline following the administration of study drugs, with only slight increases after L&I as well as after pin insertion. However, the values remained within normal limits throughout the study (±20% of baseline values).

**Figure 2:**
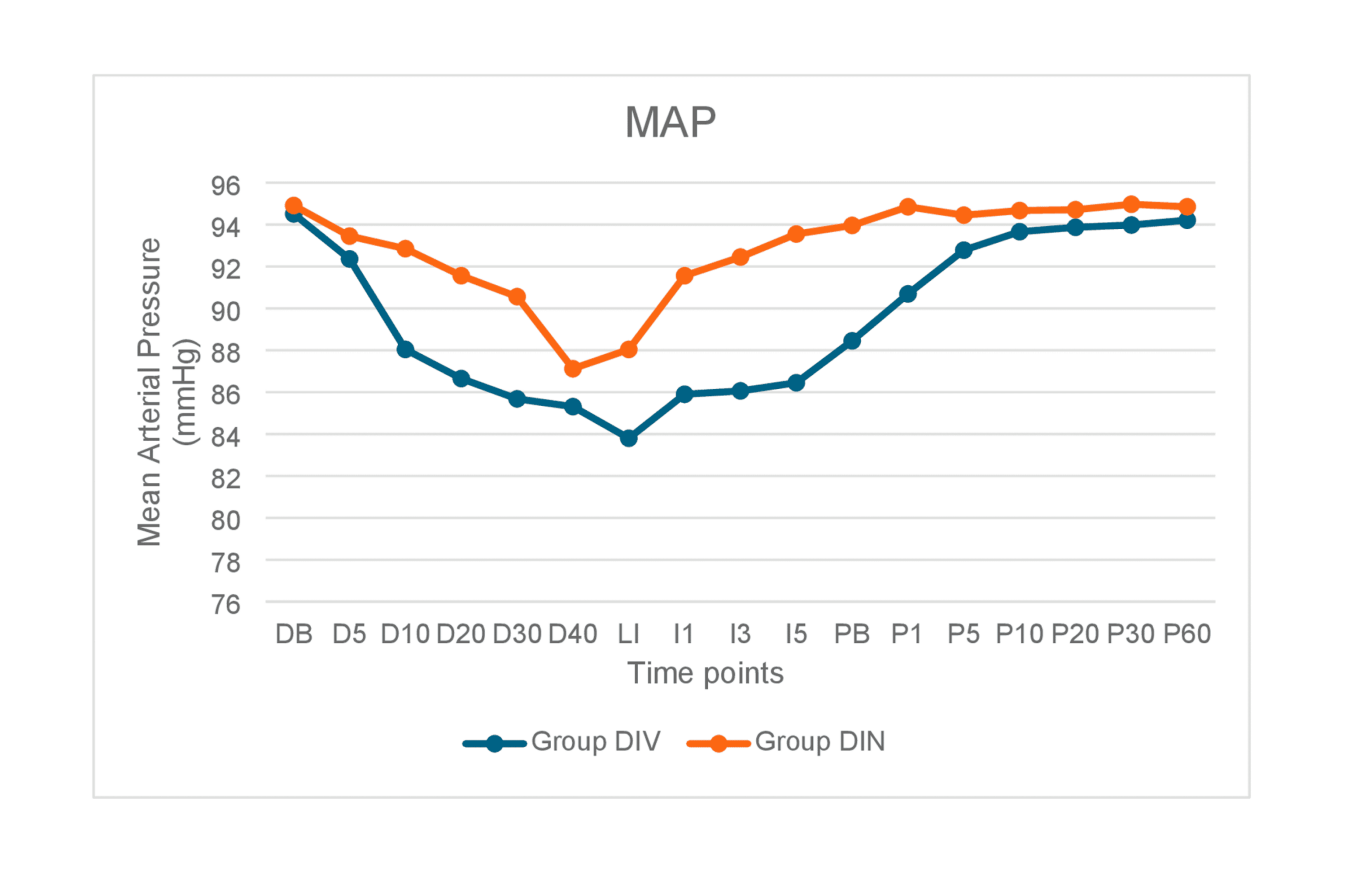
Intergroup comparison of mean arterial pressure at different time intervals.

The mean MAP was lower in Group DIV than in Group DIN at all points in time (Figure 2). The difference in mean MAP between the groups was statistically significant at all time intervals except at 5 and 40 minutes after drug administration (D5, D40), and at 10 minutes after pin insertion (P10) and thereafter. The differences in mean SBP and mean DBP between the groups were also statistically significant at most of the time intervals.

**Figure 3:**
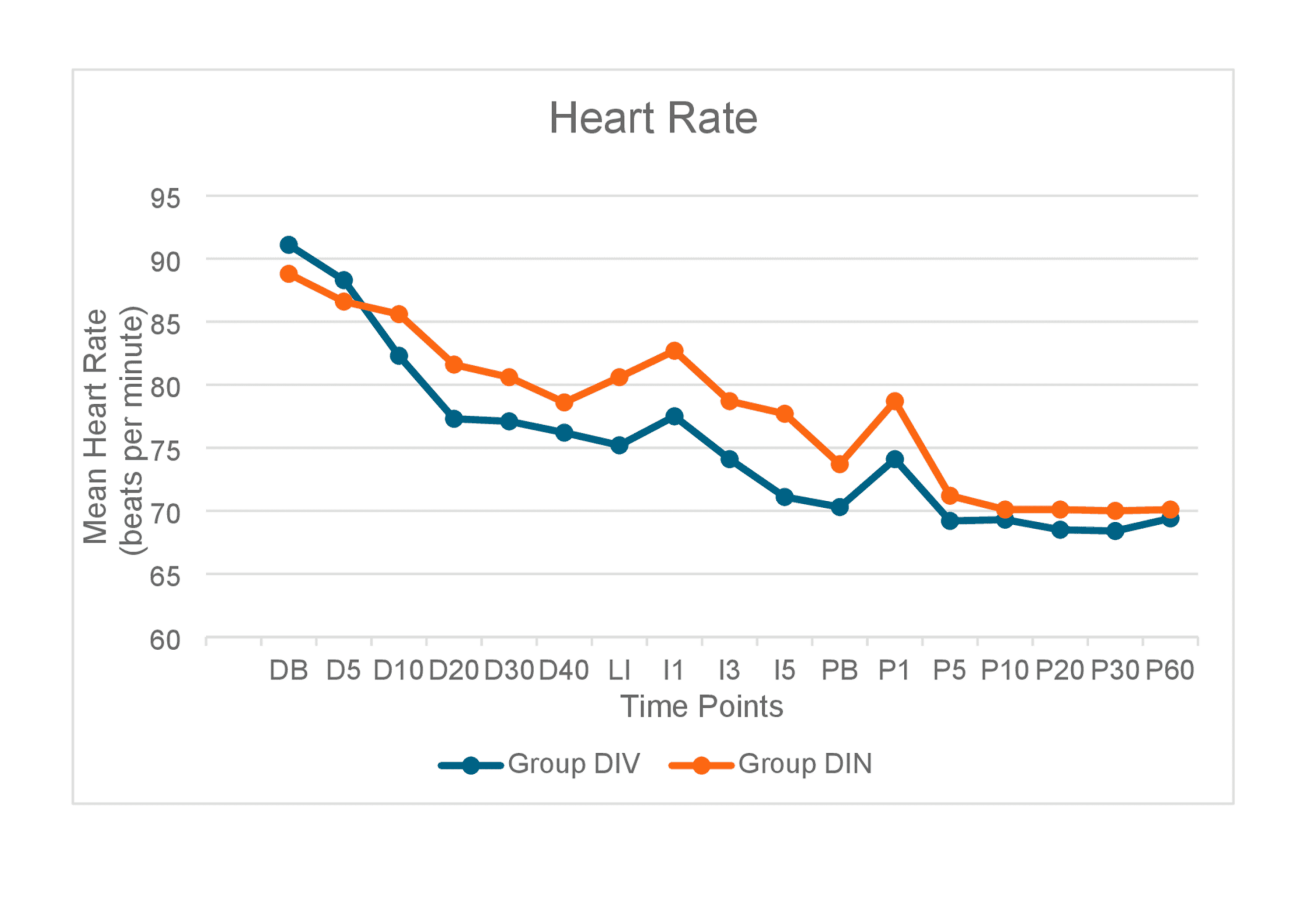
Intergroup comparison of mean heart rate at different time intervals.

The mean HR was lower in Group DIV than in Group DIN at all time points except at baseline and D5 (Figure 3). The difference in mean HR between the groups was statistically significant at all time intervals except at 5 minutes after drug administration (D5) and at 10 and 60 minutes after pin insertion (P10, P60). The incidence of hypotension and bradycardia was significantly more in the IV group than the IN group (p<0.05).

During intergroup comparisons, the RSS sedation score was significantly higher in the IV group than in the IN group at 10, 20, and 30 minutes (S10, S20, and S30) of drug administration (p<0.05) (Table 2). At these time intervals, the mean sedation score was significantly higher from baseline in the intravenous group, whereas in the intranasal group, the scores were comparable with baseline at all time points.

**Table 2:** Intergroup comparison of sedation status (RSS score) at different time intervals. (RSS score: 1 - awake; agitated or restless or both; 2 - awake; cooperative, oriented, and tranquil; 3 - awake but responds to commands only; 4 - asleep; brisk response to light glabellar tap or loud auditory stimulus; 5 - asleep; sluggish response to light glabellar tap or loud auditory stimulus; 6 - asleep; no response to glabellar tap or loud auditory stimulus). Data expressed as mean±SD; n=number of cases; %=percentage of cases; paired 't' test; *independent 't' test; p-value<0.05 was considered significant.

Time Intervals	Group DIV (n=60)	P-value (baseline to level)	Group DIN (n=60)	P-value (baseline to level)	P-value* (group DIVand DIN)
S_B_	2.6±0.8	---	2.3±1.1	---	0.0902
S_10_	4.0±0.7	<0.0001	2.1±0.5	0.2023	0.0001
S_20_	3.4±0.6	<0.0001	2.0±0.5	0.0569	0.0001
S_30_	3.1±0.5	<0.0001	2.4±0.4	0.5094	0.0001
S_40_	2.8±0.6	0.1240	2.6±0.7	0.0773	0.0955

The incidence of hypotension and bradycardia was significantly higher in the IV group compared to the IN group (Table 3). None of our patients experienced any episodes of tachycardia, respiratory depression, or nausea/vomiting during the study period.

**Table 3:** Intergroup comparison of adverse events. Data expressed as frequency; n=number of cases; chi-square test; p-value < 0.05 was considered significant.

Adverse events	Group DIV (n=60)	Group DIN (n=60)	P-value
Hypotension	7 (11.7)	3 (5.0)	0.048
Bradycardia	6 (10.0)	1 (1.7)	0.043
Hypoxia	1 (1.7)	0 (0.0)	0.315

## Discussion

Attenuation of sympathetic stress responses to L&I and to skull-pin fixation is one of the major challenges faced by anesthesiologists. Preoperative dexmedetomidine has a well-established role in attenuating stress responses to L&I.

Dexmedetomidine is a centrally acting α2-agonist, widely used for its unique sedative, analgesic, anxiolytic, sympatholytic, and antisecretory properties. It is devoid of respiratory depression and produces unique dose-dependent conscious/cooperative sedation. It is especially useful for neurosurgical patients as it allows early recovery and neurological assessment in the immediate postoperative period [18]. Due to all these characteristics, dexmedetomidine has become a popular agent for premedication.

The main disadvantages of IV dexmedetomidine are that it produces bradycardia and hypotension, and its sedative effect is more pronounced than its analgesic effect. Moreover, its rapid IV infusion may cause profound hemodynamic instability [19]. Many alternative routes of dexmedetomidine are under trial to avoid these side effects. Nowadays, the use of dexmedetomidine through the IN route for premedication is becoming increasingly popular, especially in the pediatric population. As nasal mucosa has high vascularity, dexmedetomidine may bypass the first-pass metabolism of the liver and reach the systemic circulation rapidly [14]. IN drug delivery can directly reach the central nervous system by penetrating the blood-brain barrier [20]. Besides, the IN route also obviates the need for any IV infusion and is painless, odorless, and tasteless.

In the present study, we evaluated the effects of IV and IN dexmedetomidine on the pressor responses to L&I as well as to skull-pin insertion.

The side effects of IV dexmedetomidine are mainly dose-dependent. To avoid excessive sedation and adverse hemodynamic effects, we used the effective lower dose of IV dexmedetomidine (0.5µg/kg). This dose was based on a previous study by Niyogi S et al., conducted in 2019, in which IV dexmedetomidine (0.5 µg/kg) given as an infusion over 40 minutes was effective in attenuating the stress response of L&I in patients undergoing lumbar spine surgery without any significant adverse effects [17].

The dose of IN dexmedetomidine in our study was based on a previous study by Wang SS et al., in which 1 µg/kg of IN dexmedetomidine considerably attenuated the hemodynamic response to L&I [21]. This same dose of IN dexmedetomidine has also been used recently by Hrishi PA et al., in their study in which it attenuated the increase in MAP during L&I, produced lesser hemodynamic fluctuation and provided a good surgical field during trans-nasal trans-sphenoidal skull base surgery [22].

In the present study, both IV and IN dexmedetomidine were effective in attenuating the hemodynamic responses to L&I as well as to skull-pin insertion without significant hypertension and tachycardia. All hemodynamic parameters (MAP, SBP, DBP, and HR) in both groups remained within normal limits (±20% of baseline values) before and after L&I as well as before and after skull-pin fixation. This could be due to the fact that both IV and IN dexmedetomidine prevent any rise in central catecholamine level. Dexmedetomidine inhibits the release of noradrenaline and acts on presynaptic central α2-receptors in the locus caeruleus to cause hypnosis and sedation. It prevents tachycardia and hypertension through its action on postsynaptic α2-receptors. In our study, both IV and IN dexmedetomidine could successfully attenuate the stress responses to L&I as a result of this sympatholytic property.

Findings similar to our study were seen in a study conducted by Kondavagilu SR et al., who found that a low dose of IV dexmedetomidine (0.5 µg/kg) attenuated the hemodynamic response at skull-pin insertion when compared to placebo (normal saline) [23]. Our study findings are in agreement with those of Jadhav N et al., who conducted a study in 2017 to assess the effect of IV dexmedetomidine (bolus of 1 µg/kg plus infusion of 0.5 µg/kg/min) on intraoperative hemodynamic stability in patients undergoing craniotomy [24]. They found that all hemodynamic parameters were significantly lower in the dexmedetomidine group as compared to placebo. Mahajan L et al. conducted a study to evaluate the attenuation of pressor response to L&I by IV dexmedetomidine (1 µg/kg) and IV magnesium sulfate (30 mg/kg) as compared to placebo (normal saline) [1]. IV dexmedetomidine effectively suppressed the hemodynamic response to L&I in their study. Prasad SR et al. conducted a study to compare the hemodynamic parameters and efficacy of sedation between IV dexmedetomidine (0.5 µg/kg/hr) and fentanyl (1µg/kg/hr) in pediatric cardiac surgical patients [25]. They found a decrease in HR in both groups which was less than 10-15% of baseline and did not require any intervention. This finding is similar to our study in which there was a decrease in HR in both groups which was statistically significant but clinically insignificant.

Niyogi S et al. compared the efficacy of IV dexmedetomidine (0.5 µg/kg) and IN dexmedetomidine (1 µg/kg) in attenuating the stress response of L&I in patients undergoing lumbar spine surgery [17]. Similar to our study, they also found relatively lower BP and HR in the IV dexmedetomidine group than in the IN dexmedetomidine group at all time intervals. As in our study, the difference in HR was statistically significant at all intervals. However, unlike our study, there were no statistically significant differences in MAP, SBP, and DBP at all intervals. This difference could be due to a different patient population in their study (lumbar spine surgery) compared to ours (craniotomy).

In our study, IN dexmedetomidine (1mg/kg) effectively attenuated the hemodynamic responses after L&I as well as after skull pin insertion. Similar findings were seen in the study conducted by Wang SS et al., which concluded that IN dexmedetomidine administered 30 min before induction considerably attenuates the intubation response [21]. Our results are also in agreement with those of Lu C et al., who conducted a study in 2016 and found that hemodynamic response to L&I was attenuated by IN dexmedetomidine premedication [26].

Besides the hemodynamic parameters, our study also evaluated sedation status in both groups, which was assessed by an observer using the RSS score and was significantly higher in the IV group than the IN group at 10, 20, and 30-minute intervals after study drug administration.

Sebastian B et al. conducted a study to compare IV dexmedetomidine at 0.75 mg/kg and 0.5 mg/kg with placebo (normal saline) [12]. They reported higher sedation scores in the IV dexmedetomidine group than in the normal saline group. Similarly, Prasad SR et al. conducted a study to compare the efficacy of sedation and hemodynamic parameters between IV dexmedetomidine (0.5 mg/kg) and fentanyl (1 mg/kg) in pediatric cardiac surgical patients [25]. Like our study, they also found significantly higher sedation in the IV dexmedetomidine group.

Elshafeey A et al. compared the efficacy of IN dexmedetomidine (2 mg/kg) versus IN ketamine (5 mg/kg) for anxiolysis and sedation before pediatric general anesthesia, 30 minutes before the procedure [27]. They reported statistically significant sedation in the dexmedetomidine group compared to the ketamine group. Barends CRM et al. conducted a study to determine the safety and tolerability of IN dexmedetomidine in the elderly (>65 years) with or without beta-blockade [28]. They found IN dexmedetomidine in the elderly had a sedative effect which is somewhat similar to our study in which IN dexmedetomidine also produces some sedation effects, however, this is much less compared to IV dexmedetomidine.

As stated earlier, Niyogi S et al. conducted a study to compare IV dexmedetomidine (0.5 mg/kg) and IN dexmedetomidine (1 mg/kg) for their efficacy in attenuating the stress response of L&I in patients undergoing lumbar spine surgery [17]. They also observed a similar change in sedation as it was in our study.

In our study, adverse events like bradycardia and hypotension were experienced more frequently in the IV group than in the IN group. Sebastian B et al. conducted a study to compare IV dexmedetomidine at 0.75 mg/kg and 0.5 mg/kg with placebo (normal saline) [12]. Unlike our study, none of the patients in their study had any adverse effects such as bradycardia, hypotension, respiratory depression, and a fall in oxygen saturation.

Our study had some limitations. It was a single-center study with a limited number of subjects. As we restricted our study to ASA I and II patients, the results cannot be extrapolated to sicker neurosurgical patients with higher ASA grades. The assessment of sedation was done using the RSS score, which is subjective and prone to bias. Monitoring plasma dexmedetomidine and catecholamine levels could have provided better comprehension and more insight into the usefulness of dexmedetomidine.

## Conclusions

In this study, dexmedetomidine effectively attenuated the hemodynamic stress response to L&I and skull-pin fixation without causing significant hypertension or tachycardia, whether administered intravenously (0.5 µg/kg) or intranasally (1 µg/kg) in patients undergoing craniotomy. However, intranasal dexmedetomidine caused fewer sedative effects and adverse effects than intravenous dexmedetomidine. Therefore, we concluded that IN dexmedetomidine can be used as an alternative to IV dexmedetomidine for blunting the hemodynamic responses to L&I and to skull-pin fixation in patients undergoing elective craniotomy, and it is relatively safer, causing less sedation and fewer side effects. However, larger multicentric studies are needed to validate the efficacy of IN dexmedetomidine in these patient populations and procedural settings. In the future, similar studies could include added monitoring of plasma dexmedetomidine levels and plasma catecholamine levels to provide better comprehension and more insight into the usefulness of dexmedetomidine in various clinical settings.
